# Damage Detection of Regular Civil Buildings Using Modified Multi-Scale Symbolic Dynamic Entropy

**DOI:** 10.3390/e24070987

**Published:** 2022-07-17

**Authors:** Tzu-Kang Lin, Dong-You Lee, Yu-Chung Hsu, Kai-Wei Kuo

**Affiliations:** Department of Civil Engineering, National Yang Ming Chiao Tung University (NYCU), Hsinchu 300, Taiwan; icrtqweasd19@gmail.com (D.-Y.L.); andrew08300000@gmail.com (Y.-C.H.); a1055247@gmail.com (K.-W.K.)

**Keywords:** structural health monitoring, multi-scale, dynamic symbol entropy, ambient vibration

## Abstract

Based on the examination of the fundamental characteristics of structures, structural health monitoring (SHM) has received increased attention in recent years. Studies have shown that the SHM method using entropy analysis can precisely identify the damaged location of the structure, which is very helpful for the daily inspection or maintenance of civil structures. Although entropy analysis has shown excellent accuracy, it still consumes too much time and too many resources in terms of data processing. To improve the dilemma, in this study, modified multi-scale symbolic dynamic entropy (MMSDE) is adopted to identify the damaged location of the civil structure. A damage index (DI) based on the entropy diagram is also proposed to clearly indicate the damage location. A seven-story numerical model was created to verify the efficiency of the proposed SHM system. The results of the analysis of each case of damage show that the MMSDE curve for the damaged floor is lower than that for the healthy floor, and the structural damage can be correctly diagnosed by the damage index. Subsequently, a scaled-down steel benchmark experiment, including 15 damage cases, was conducted to verify the practical performance of the SHM system. The confusion matrix was used to further evaluate the SHM system. The results demonstrated that the MMSD-based system can quickly diagnose structural safety with reliability and accuracy. It can be used in the field of long-term structural health monitoring in the near future.

## 1. Introduction

Structural health monitoring (SHM) [1,2,3] refers to the use of on-site non-destructive sensing technology, combined with structural system characteristic analysis, to detect structural damage or degradation [4,5]. According to the evaluation method, it can be divided into a destructive evaluation (DE) and a non-destructive evaluation (NDE) [6]. In general, structure information is usually recorded by sensors for non-destructive inspection, and a metamodeling scheme is then applied [7,8]. Subsequently, various algorithms and the corresponding damage index value are developed as the basis for identifying whether or not the structure is damaged [9,10,11,12]. Recently, entropy-based analysis has been widely applied in different fields. In this study, in order to provide a rapid and reliable evaluation process for unsafe and old buildings, the entropy analysis is adopted to determine whether the structure is damaged.

In 1948, Shannon applied the classic entropy concept into information theory, which was called information entropy [13]. The approximate entropy (ApEn) was proposed by Pincus et al. in 1911, on the basis of Kolmogorov entropy, which can complete the judgment of the complexity of the time series without the coarse-graining process [14]. Sample entropy (SampEn) was proposed in 2000, which improved the shortcomings of ApEn and reduced the influence of sample length on the analysis results [15,16]. Multiscale entropy (MSE) was proposed by Costa et al. in 2002 to convert the measured original signal to different scales, and then sampling entropy analysis was performed to solve the problem that single-scale sampling entropy cannot distinguish pathological signals [17,18].

In recent years, entropy has gradually received attention in the field of damage identification. Rojas used ultrasound to detect tiny perforations in plate-shaped structural members and discussed the optimal configuration of the sensor [19]. In 2017, Wimarshana et al. used wavelet transformation and sampling entropy to detect breathing cracks on a cantilever beam and optimized the sampling entropy parameters [20]. After the optimization process, the detection efficiency was improved. Guan et al. applied cross-entropy to the failure detection of shear structures and used the finite element model, with input white noise, to simulate random loads, successfully verifying the feasibility of this system [21]. In the mechanical field, many methods have made important contributions to the fault monitoring of planetary gears, including fault feature extraction [22,23]. In 2015, Wang et al. [24] proposed symbolic dynamics entropy (SDE), which combines the advantages of dynamic symbols and information theory to extract the fault features of an aviation electro-mechanical actuator. After conducting simulations and tests, it was found that SDE not only overcomes the shortcomings of the sample entropy of poor computational efficiency in the event of longer time series, but also inherits their advantages. However, this method has two major drawbacks. First, this method only considers the state mode probability and does not consider the relationship between state transitions. Second, this method only considers the state at a single scale, which limits the relatively complex analysis capability of vibration modes. Therefore, Li et al. proposed a modified multi-scale symbolic dynamic entropy (MMSDE) in 2016, which overcomes the shortcomings of SDE, and applied it to the health monitoring of planetary gears [25]. As the MMSDE analysis has been proven to have good performance for fault detection in mechanical engineering, it is adopted in this study. Moreover, a novel damage index based on the MMSDE diagram is proposed to jointly identify the damage location of the structure.

**Figure 1:**
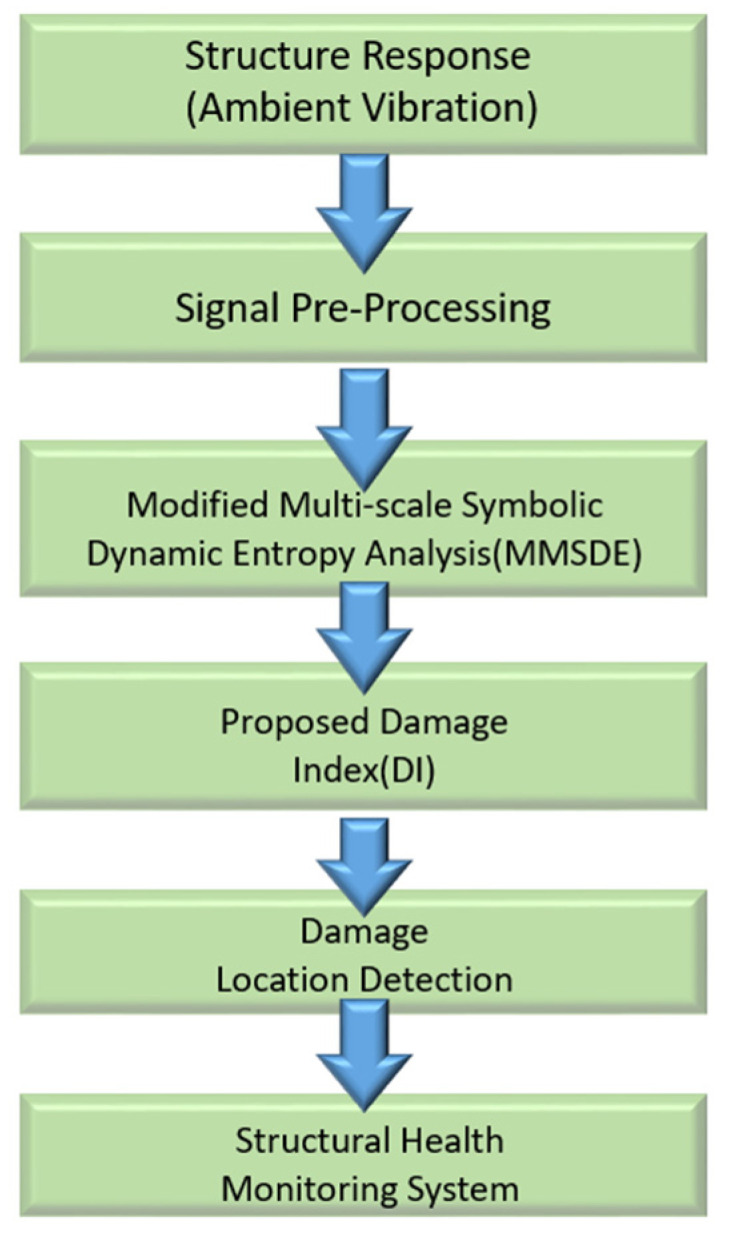
Flow chart of the proposed SHM system.

For the high performance of detecting the failure mode of planetary gears in the mechanical field, the MMSDE analysis is adopted to identify the possible damage existing in building structures. The velocity signals of each floor of the structure under ambient vibration are collected and analyzed by MMSDE with the proposed damage index. The remainder of this study is structured as follows. The methodology, including modified multi-scale symbolic dynamic entropy and the proposed damage index, is introduced first. In Section 3, a numerical model of the seven-story unidirectional stiffened steel frame is constructed. The MMSDE curves and the damage index are applied to 16 damage cases to demonstrate the feasibility of the proposed method. To verify the practicality of the SHM system, an ambient vibration experiment for a scaled-down steel benchmark structure was conducted in Section 4. The confusion matrix was utilized to evaluate the practical performance of the system. Finally, a summary is provided, and conclusions are drawn. The flow chart of the proposed SHM system is shown in Figure 1.

## 2. Methodology

### 2.1. Symbolic Dynamic Entropy (SDE)

The dynamic symbol entropy analysis is composed of four steps. First, the time series $\left\{X_{i}\right\}=\left\{x_{1},\;\ldots ,x_{i},\ldots ,x_{N}\right\}$ is converted to a symbol series $\left\{Z_{i}\right\}=\left\{z_{1\;},\;\ldots ,z_{i\;},\ldots ,z_{N}\right\}$ (symbolization). The maximum entropy partitioning (MEP) is used to divide the range into *ε* intervals. The values of the original time series are rearranged, from the smallest to the largest, to find the value corresponding to the rearranged time series with an integer value less than or equal to *N/ε*. Finally, the partitions of the MEP are compared, and the corresponding symbols are converted to complete the symbolization.

According to the symbol sequence, the embedding vectors changed with m are established, and the probability of potential state patterns is calculated. For different embedding dimensions (m) and time delays (*λ*), the symbol sequence can be expressed as:

(1) $$Z_{j}^{m,\lambda }\left\{z\left(j\right),z\left(j+\lambda \right),\ldots ,z\left(j+\left(m-1\right)\lambda \right)\right\},\;j=1,2,\ldots ,N-\left(m-1\right)\lambda$$

where $Z_{j}^{m,\lambda }$ have m units, each unit has *ε* symbols, and the subvectors are independent.

There are a total of $\varepsilon^{m}$ potential state modes. Subsequently, the state of the embedding vector is expressed as $q_{a}^{\varepsilon ,m,\lambda }\;\left(a=1,2,\ldots ,\varepsilon^{m}\right)$. The probability of potential state mode $P(q_{a}^{\varepsilon ,m,\lambda })$ can be derived as:

(2) $$P\left(q_{a}^{\varepsilon ,m,\lambda }\right)=\frac{\left\|\{j:j\leq N-\left(m-1\right)\lambda ,type\left(Z_{j}^{\varepsilon ,m,\lambda }\right)=q_{a}^{\varepsilon ,m,\lambda }\}\right\|}{N-\left(m-1\right)\lambda }$$

The symbolized time series contains a finite number of states in the series. As time progresses, it transitions to the next state (including self-circulation). This step is to calculate the conditional probability of the state mode transition over time. The original state mode is expressed as: $q_{a}^{\varepsilon ,m,\lambda }\left(a=1,2,\ldots ,\varepsilon^{m}\right)$, and the next symbol that appears is expressed as $\sigma_{b}\left(b=1,2,\ldots ,\varepsilon \right)$. Then, the conditional probability transition can be expressed as:

(3) $$P\left(\sigma_{b}|q_{a}^{\varepsilon ,m,\lambda }\right)=P\left\{z\left(j+m\lambda \right)=\sigma_{b}|j:j\leq N-m\lambda ,\mathrm{type}\left(Z_{a}^{\varepsilon ,m,\lambda }\right)\right\}$$

According to the above formula, Equation (3) can be rewritten as:

(4) $$\left(\sigma_{b}|q_{a}^{\varepsilon ,m,\lambda }\right)=\frac{\left\|\{j:j\leq N-m\lambda ,type\left(Z_{j}^{\varepsilon ,m,\lambda }\right)=q_{a}^{\varepsilon ,m,\lambda },z\left(j+m\lambda \right)=\sigma_{b}\}\right\|}{N-m\lambda }$$

Finally, based on Shannon’s entropy theory, the symbolic dynamic entropy SDE(X,m,λ,ε) is defined as the sum of state entropy and the transition of entropy, which can be expressed as:

(5) $$SDE\left(X,m,\lambda ,\varepsilon \right)=-\overset{\varepsilon^{m}}{\underset{a=1}{\sum }}P\left(q_{a}^{\varepsilon ,m,\lambda }\right)\cdot lnP\left(q_{a}^{\varepsilon ,m,\lambda }\right)-\overset{\varepsilon^{m}}{\underset{a=1}{\sum }}\overset{\varepsilon }{\underset{b=1}{\sum }}P\left(q_{a}^{\varepsilon ,m,\lambda }\right)\cdot ln\left(P\left(q_{a}^{\varepsilon ,m,\lambda }\right)\cdot P\left(\sigma_{b}|q_{a}^{\varepsilon ,m,\lambda }\right)\right)$$

The symbolic dynamics entropy was proposed to collect the fault characteristics of aviation electromechanical actuators [26]. Only the probability of the state mode is considered in the method. However, the conditional probability, which indicates the frequency of transition from one state to another, is also important for extracting damage information. In this study, the multi-scale symbol dynamic entropy (MMSDE) proposed by Li [27] is used to extract more comprehensive damage features.

### 2.2. Modified Multi-Scale Symbolic Dynamic Entropy (MMSDE)

According to the multi-scale analysis method proposed by Costa, the dynamic characteristics of different time series can be evaluated more accurately. However, the conventional multi-scale analysis using the coarse-grained process shortens the original length of the time series, and the irregular fluctuations in the entropy value may be generated under the original SDE method. An improved multi-scale analysis method, which uses the moving averaging procedure to replace the coarse-grained process, was proposed by Wu [28] et al. By introducing the moving averaging procedure, the original SDE was improved into a modified multi-scale symbolic dynamic entropy (MMSDE). More template vectors can be generated from the original time series, which allow MMSDE to provide a greater estimation of accurate entropy.

The moving average procedure can be completed by processing the time series $\left\{X_{i}\right\}=\left\{x_{1\;},\;\ldots ,x_{i\;},\ldots ,x_{N}\right\}$ as:

(6) $$y_{j}^{\tau }=\frac{1}{\tau }\overset{j+\tau -1}{\underset{i=j}{\sum }}x_{i}\;\;\;\;1\leq j\leq N-\tau +1$$

where τ is the scale factor.

Then, the time series $\left\{y_{j}^{\tau }\right\}$ under each scale is calculated by the above-mentioned procedure of modified SDE. Finally, the graph can be plotted as a function of the scale factor. $MMSD\left(x,\tau ,m,\lambda ,\varepsilon \right)=SDE\left(y_{j}^{\tau },m,\lambda ,\varepsilon \right).$ This procedure is called MMSDE.

The SDE process involves three parameters, including the embedding dimension m, time delay λ, and quantity symbol ε. To find the best parameters, the average Euclidean distance (AED) is used to select the best parameters. AED is defined by calculating the Euclidean distance (ED) between different conditions using SDE values. The detailed process of AED is briefly described as follows. First, suppose that the given data have k different classes and each class has n samples of length L. Then, initialize the parameters m, *ε* in SDE and set *ε* ∊ [2,20]; m is set according to $\varepsilon^{m}$ < L. Calculate the SDE value of each sample *i*th class, and calculate the Euclidean distance (ED) between the *i*th and *j*th classes. A larger AED value means that the conditions are more distinguishable, which means that the SDE has a better ability to extract useful information from the vibration signals. The definition of ED and AED is expressed as:

(7) $$ED\left(i,j\right)=\sqrt{\sum_{p-1}^{n}{\left(SDE_{i}\left(p\right)-SDE_{j}\left(p\right)\right)}^{2}}$$

(8) $$AED=\overset{k}{\underset{i=1,j=1}{\sum }}\overset{k}{\underset{j\neq i}{\sum }}ED\left(i,j\right)$$

**Figure 2:**
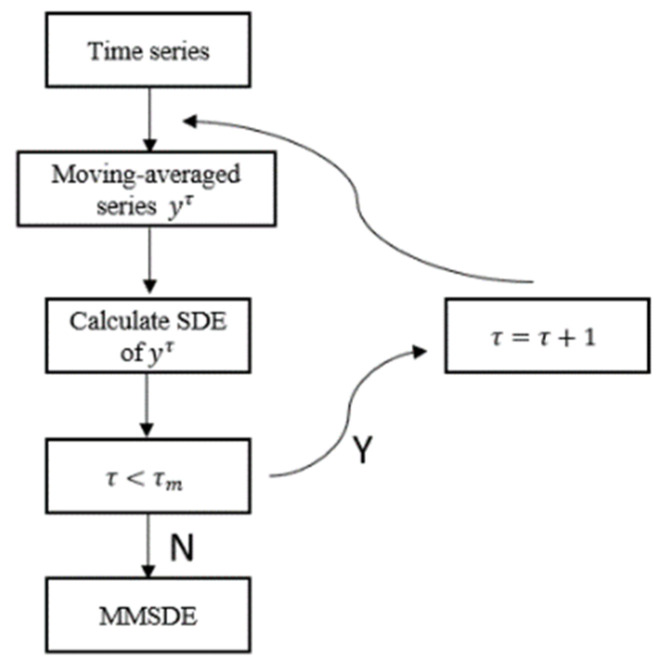
The MMSDE flow chart.

The AED value is calculated by updating the parameters and repeating the steps. By selecting the optimal parameters based on the higher value of the AED, the optimal setting of the parameters can be evaluated. In this study, time delay has little effect on the results, and λ is set to 1. The MMSDE flow chart is shown in Figure 2.

### 2.3. Damage Index

The damage index proposed in the previous research is used to quantify the degree of similarity and non-synchronization between the signals, as an indicator for the detection of damaged floors [29]. The severity of damage was successfully assessed by a damage quantification system based on composite multiscale cross-sample entropy [30]. However, in this paper, due to the influence of the on-site noise, only the damage location identification is considered. The damage index is expected to reduce the probability of error caused by human judgment. The method of the damage index is to calculate the entropy of damaged buildings minus the entropy of undamaged buildings on the same floor, and then add up the values of the individual floors. In this paper, considering the characteristics of MMSDE, the damage index is revised to subtract the area of the entropy curve of adjacent floors under different scales to accurately determine the damaged floor.

In health and damage cases, the entropy curve $E_{F}\;\left(F=1,2,\ldots 7\right)$ of each floor can be subtracted from those of the upper and lower floors to obtain the area difference $A_{\tau }^{H},A_{\tau }^{D}$, which can be expressed as:

(9) $$A_{\tau }^{H}=E_{F}^{H}-E_{F-1}^{H}\;\;\;\;\;\;\;\;\;\;\;\;\;\;A_{\tau }^{D}=E_{F}^{D}-E_{F-1}^{D}$$

The equation represents the area of the entropy curve under the health condition and the damaged condition of different floors, respectively. Accordingly, the damaged index (D*I*) can be obtained as

(10) $$DI_{F}=\overset{10}{\underset{\tau =1}{\sum }}\left(A_{\tau F}^{H}-A_{\tau F}^{D}\right)$$

A threshold γ was applied to the calculated the DI value to enhance the identification result. When the calculated value of the damage index is more than γ, the floor is determined as damaged; conversely, the floor is not damaged when the damage index is smaller than the preset threshold. As mentioned above, the threshold was determined as 2. When $DI_{F}>\gamma$, the floor is determined as damaged; conversely, when $DI_{F}\leq \gamma$, the floor is not damaged.

## 3. Numerical simulation

### 3.1. Database of Numerical Simulation

**Figure 3:**
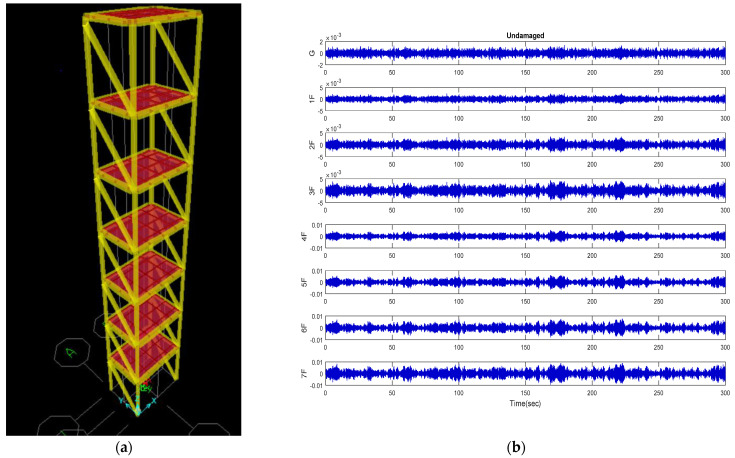
(**a**) Three−dimensional seven-story numerical model; (**b**) velocity response under ambient vibration (undamaged case).

The numerical model of the seven-story unidirectional stiffened steel frame is built as shown in Figure 3. The material property is A36 steel, and the yield strength $f_{y}$ is 2500 kg/c$m^{2}$. The numerical model is simulated with beams, columns, and plates. The column section measures 25 mm × 150 mm, the height of each floor is 1.06 m, and the angle steel bracing section size is 65 mm × 65 mm × 6 mm. The beam section measures 100 mm × 70 mm. The long side of the floor slab is 1.32 m, and the short side is 0.92 m. The damping ratio of the structure is set to 0.02. To simulate the ambient vibration signal of environmental disturbance, white noise with the output energy of one milliwatt (0 dBm) was used as input. The data time interval of each point was 0.005 s, and the total length of the signal was 5 min.

The numerical simulation, divided mainly into 19 cases, is shown in Table 1. Due to the fluency and simplicity of the experiment execution, the damage floors were set to be adjacent floors in this study. The degree of damage is categorized as follows: (1) undamaged: healthy state of the structure (bracings are all installed), (2) one-story damaged: single-story bracing removed, (3) two-story damaged: two-story bracings removed, and (4) three-story damaged: the bracing from three adjacent floors removed.

### 3.2. MMSDE of Numerical Simulation

After a variety of parameter tests, the embedding dimension (m) is selected as 3, the time series are converted into sequences using MEP, and the number of converted symbols ε is set to 10. The relevant study of MMSDE shows that the best range of event data length is $\varepsilon^{m}<L$; therefore, the sample length is set at 500 points. The damage analysis is carried out with this parameter setting.

**Figure 4:**
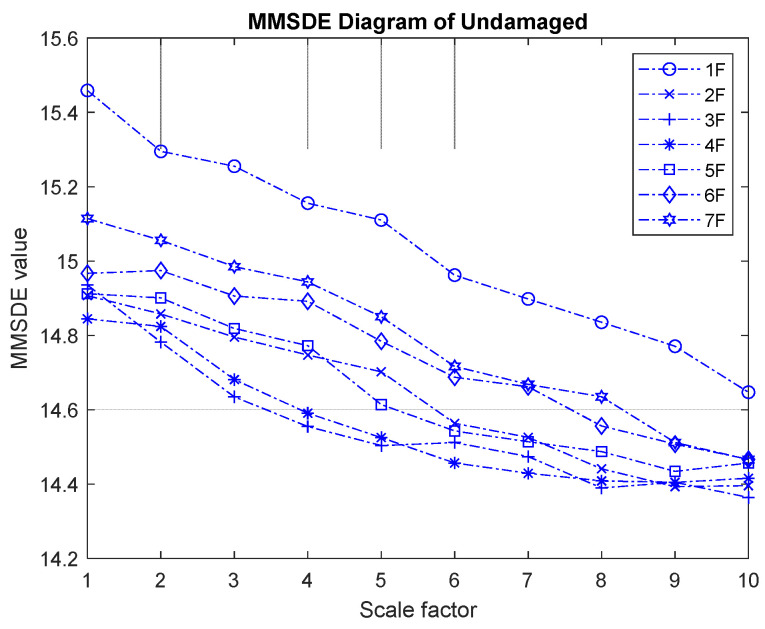
MMSDE diagram of the undamaged case.

**Figure 5:**
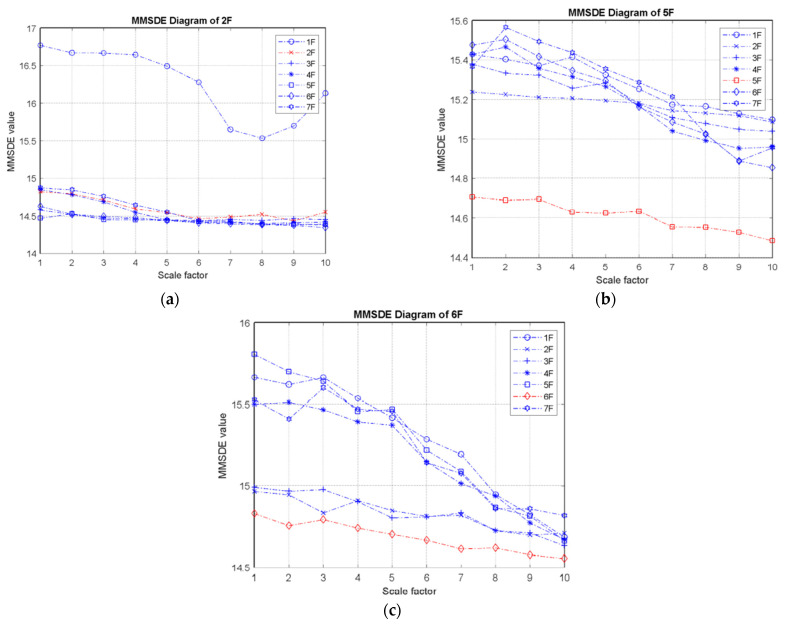
MMSDE diagram of single-story damage cases; (**a**) second floor damaged; (**b**) fifth floor damaged; (**c**) sixth floor damaged.

**Figure 6:**
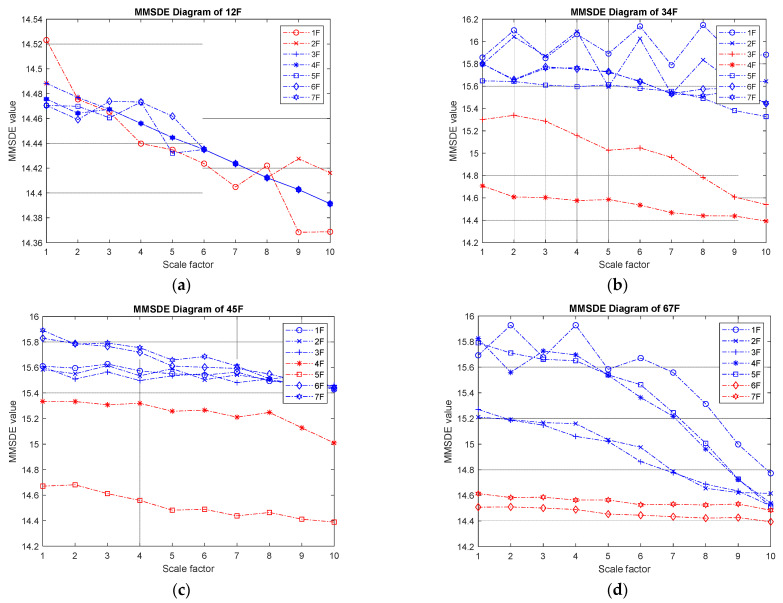
MMSDE diagram of two-story damage; (**a**) first and second floors damaged; (**b**) third and fourth floors damaged; (**c**) fourth and fifth floors damaged; (**d**) sixth and seventh floors damaged.

**Figure 7:**
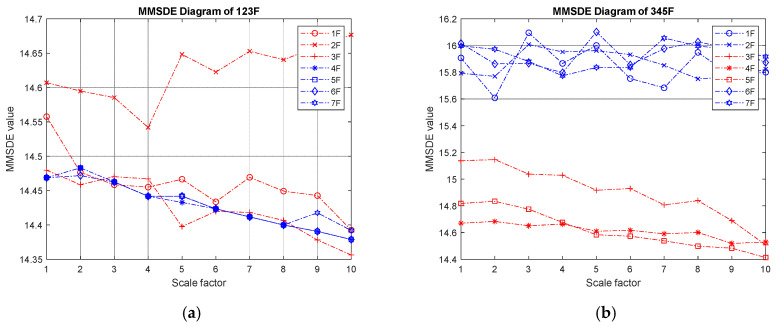

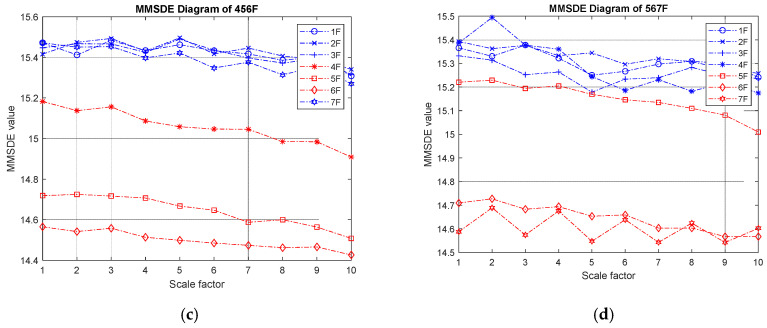
MMSDE diagram of three-story damage; (**a**) first, second, and third floors damaged; (**b**) third, fourth, and fifth floors damaged; (**c**) fourth, fifth, and sixth floors damaged; (**d**) fifth, sixth, and seventh floors damaged.

The MMSDE entropy curve of the seven-story building under the undamaged condition is used as a reference for other cases to evaluate the damage condition. Figure 4 shows the MMSDE curve for the undamaged case of the model. Under the healthy condition, the trend of the entropy curve on different floors is quite similar. Not much fluctuation is observed, and the entropy gradually decreases as the scale increases.

(I) One-story damage: Figure 5 shows the MMSDE entropy curve for the single-story damaged condition. It can be observed that when the single-story bracing is removed, the entropy curve of this floor decreases, and the entropy values of other healthy floors also increase. For the case of the second floor damage, shown in Figure 5a, the structural failure affects the entropy value of the other adjacent floors, causing the entropy of the first floor to decrease. From the entropy curves in Figure 5b,c, the reduction in the entropy value of the damaged floor can be clearly seen. The maximum value of the entropy curve of the damage on the fifth floor is reduced to approximately 14.7, and the damage on the sixth floor is reduced to approximately 14.8.

(II) Two-story damage: the MMSDE entropy curve for the two-story damage conditions is shown in Figure 6. The respective MMSDE curves of healthy and damaged floors are speculated to distribute in opposite directions. However, in the case of low-floor damage, it can be seen that the curve of the damaged floor and the healthy floor cannot be clearly distinguished in Figure 6a, which leads to a misjudgment. In Figure 6b,c, the maximum values of the entropy curves of the consecutive failures on the third and fourth floors and the consecutive failures on the fourth and fifth floors are reduced to less than 15.4. In Figure 6d, the maximum value of the entropy curve of the damage on the sixth and seventh floors is reduced to approximately 14.6, and a significant difference between the failure and the healthy floor curve can be observed.

(III) Three-story damage: Figure 7 shows the MMSDE entropy curve of the three-story damaged condition. In the case of low-floor damage, the MMSDE curves of the case where the first to third floors are damaged do not distribute as expected. The entropy value of the damaged floors is higher than that of the healthy floors. It is speculated that the diagnosis may be inaccurate when the lower floors are continuously damaged. In the case of continuous damage on the higher three floors, the entropy value of the damaged floor is significantly reduced. Specifically, the maximum entropy value in the case of continuous failure of the fifth, sixth, and seventh floors is reduced to 14.7.

### 3.3. Damage Index

**Figure 8:**
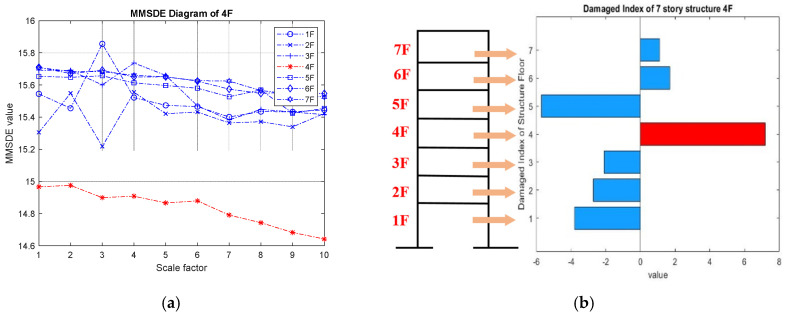
Detection result of the four-story damage case; (**a**) MMSDE curve; (**b**) damage index.

As the damaged floor cannot be clearly distinguished by the MMSDE curve, a misjudgment may occur. The damage index (DI) is proposed as a further quantitative judgment for damage detection. The results of damage on the fourth floor are shown in Figure 8a,b. In the entropy curve, except for the fourth floor, the entropy values of other floors increase compared to the reference. From Figure 8a, it can be seen that the entropy value of the healthy floor increases, but the entropy value of the damaged floor itself decreases significantly. The entropy values of the first floor and the second floor on scales 2 and 3 fluctuate significantly, but the result does not affect the judgment of the damaged floor by DI. According to the DI shown in Figure 8b, the fourth floor can be clearly determined as damaged.

**Figure 9:**
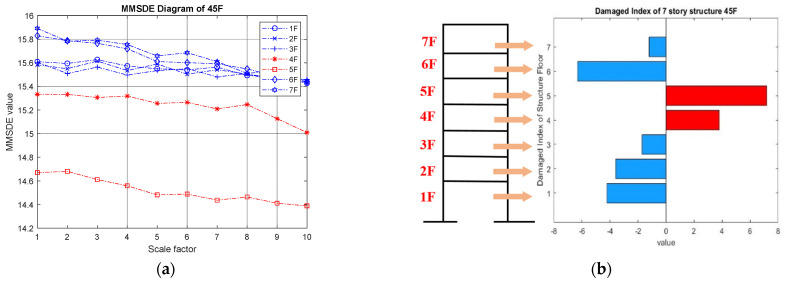
Detection result of the four- and five-story damage case; (**a**) MMSDE curve; (**b**) damage index.

The damage results of the fourth and fifth floor damage are shown in Figure 9a,b. Figure 9a shows that the curves for the fourth and fifth floors are lower than those for the undamaged floors. The entropy values of the healthy floor and the damaged floor are concentrated, respectively. Except for the entropy curve of the fifth floor, the values of the other floors are increased compared to the initial reference, while the curve of the fifth floor is not changed significantly. The damage index indicates that the fourth and fifth floors are damaged.

**Figure 10:**
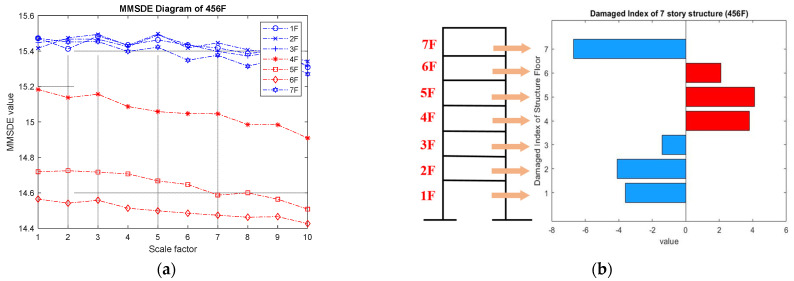
Detection result of the fourth- to the sixth-story damage case. (**a**) MMSDE curve; (**b**) damage index.

In the case of three-floor damage, Figure 10a,b illustrates the MMSDE curves and the damage index for damage on floors ranging from the fourth floor to the sixth floor. The trend of this case is similar to the result of the fourth and fifth story damage case. Only the entropy curves for the fourth, fifth, and sixth floors are lower, and the entropy values of the other undamaged floors are higher. From Figure 10b, the fourth, fifth, and sixth floors can be successfully judged as damaged by the damage index.

From the numerical analysis, the entropy curves exhibit a special trend under different damage cases. The entropy values of the damaged floors are lower than those of the undamaged floor at different scales, and the entropy curves of undamaged and damaged floors show separate aggregations in the MMSDE diagram. When the distribution trend cannot be clearly seen from the entropy diagram, the damage index defined by the MMSDE curve can be used to determine the location of the damaged floor.

### 3.4. Confusion Matrix Verification

The confusion matrix is applied to further evaluate the performance of MMSDE under different damage cases. Three evaluation indices, including accuracy, precision, and recall, are adopted, and the entries of the confusion matrix are defined as:

(11) $$Accuracy=\frac{TP+TN}{TP+TN+FP+FN}\;\;\;\;\;Precision=\frac{TP}{TP+FP}\;\;\;\;Recall=\frac{TP}{TP+FN}$$

The damage indices are divided into four categories: (1) true positive (*TP*): a damaged floor can be correctly judged as damaged by the damage index; (2) false positive (*FP*): an undamaged floor is misjudged as damaged; (3) true negative (*TN*): an undamaged floor can be successfully identified as undamaged based on the damage index; (4) false negative (*FN*): a damaged floor is misjudged as undamaged. The three evaluation parameters can be used to objectively determine the accuracy of a method. “Accuracy” represents the overall accuracy of the classifier. “Precision” refers to the proportion of floors that are classified as damaged, and are actually damaged. “Recall” is used to determine the proportion of the actual damaged floors that are correctly classified as damaged. The classification results shown in Table 2 indicate that this method is reliable for damaged floor monitoring with the appropriate setting of MMSDE parameters. The accuracy, precision, and recall rates can reach 92.8%, 90.3%, and 82.3%, respectively.

## 4. Experimental Verification

### 4.1. Experimental Database

The confusion matrix evaluation in the numerical simulation has demonstrated that the location of the damaged floor can be accurately determined by MMSDE. Therefore, the experimental verification is carried out to compare the difference of the entropy curve between the experiment and the numerical simulation. To verify the practicability of the SHM system, an ambient vibration test was conducted on a scaled-down steel reference structure. In addition, as the characteristics of the experimental structure is similar to the numerical model, the number of damage cases is designed to be the same as the numerical simulation.

**Figure 11:**
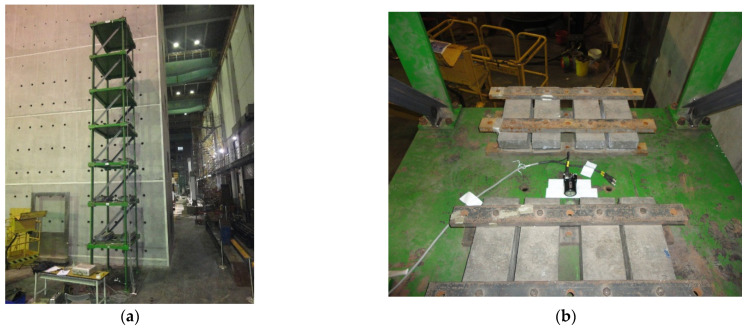
Experimental setup. (**a**) Seven-story steel frame model; (**b**) location of the sensor and mass block.

**Figure 12:**
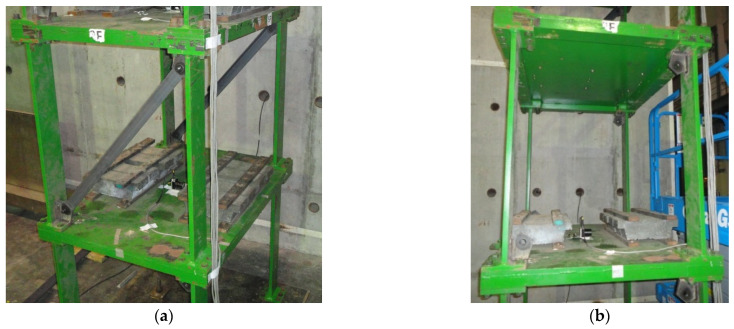
Experimental installation of destructive and non-destructive structures; (**a**) undamaged condition (no bracing removed); (**b**) damaged condition (bracing removed).

The experimental setup of the seven-story steel structure is shown in Figure 11. Each floor is a rectangular base plate of 1.5 m by 1.1 m, and the height of each floor is 1.1 m. The section size of the column is 150 mm × 25 mm. Each floor is loaded with a 500 KG mass block to simulate the actual structural characteristics. Additionally, the bracing is installed on the weak axis to simulate the damaged case. The bracing is made of 65 mm × 65 mm × 6 mm L-shaped steel, which is the same as in the numerical simulation. The damage simulation is depicted in Figure 12.

**Figure 13:**
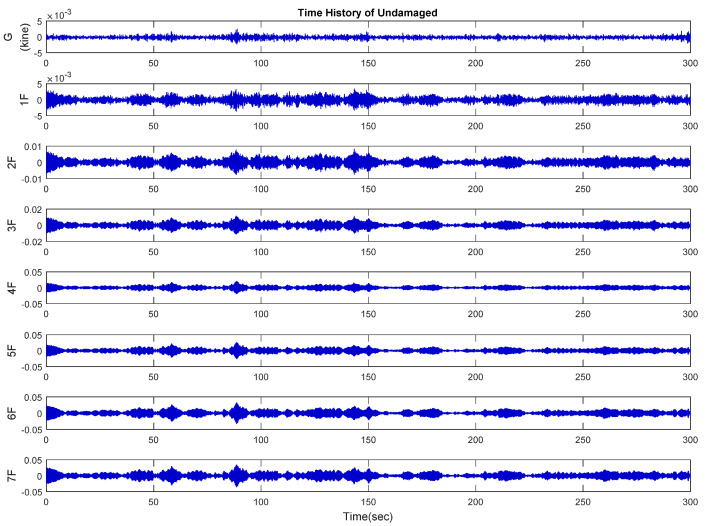
Velocity response for ambient vibration measurement (undamaged).

The VSE-15D sensor was deployed in this experiment, recording the ambient vibration from the weak axis in the center of the floor. Each damage case was measured at 200 Hz for five minutes. The velocity response signal of the case without damage is shown in Figure 13. The top-floor signal of each damage case was processed by fast Fourier analysis (FFT) to confirm the signal quality of the experimental data, and the results are listed in Table 3. As indicated, the frequency of undamaged condition is 3.34 Hz, which is the highest in the table. Due to structural damage, the fundamental frequency gradually decreased, indicating that the removal of diagonal bracing has an increasing impact on the overall rigidity and stiffness of the structure.

### 4.2. Results of Experimental Verification

**Figure 14:**
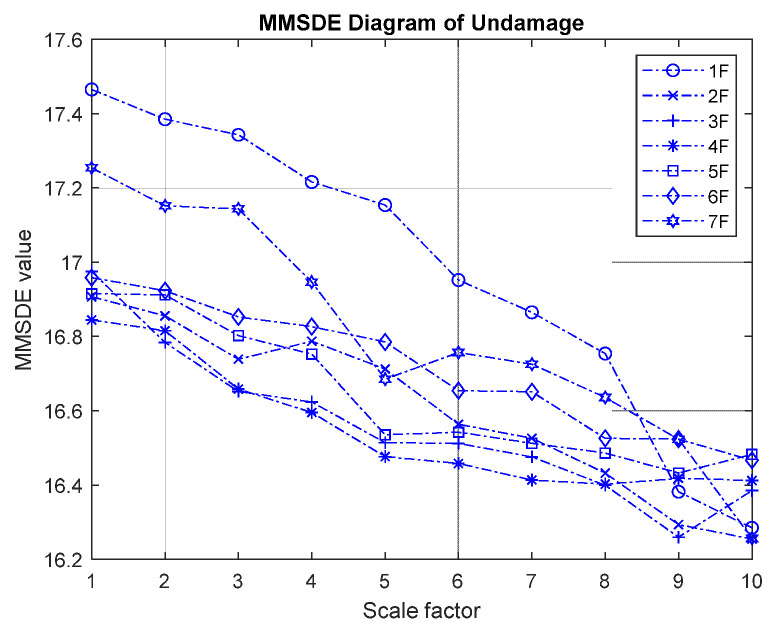
Experimental MMSDE diagram of an undamaged case.

In the experimental verification of MMSDE performance, the case that all floors are braced is used as the reference. By comparing the entropy curve of the damaged case with the reference, the damage index can be obtained. Figure 14 presents a MMSDE diagram for the undamaged condition (reference). Although the entropy value is raised slightly, the curve distribution is similar to the result of an undamaged condition in the numerical model. When the scale is higher, the entropy curve decreases slightly and gradually converges.

(I) One-story damage

**Figure 15:**
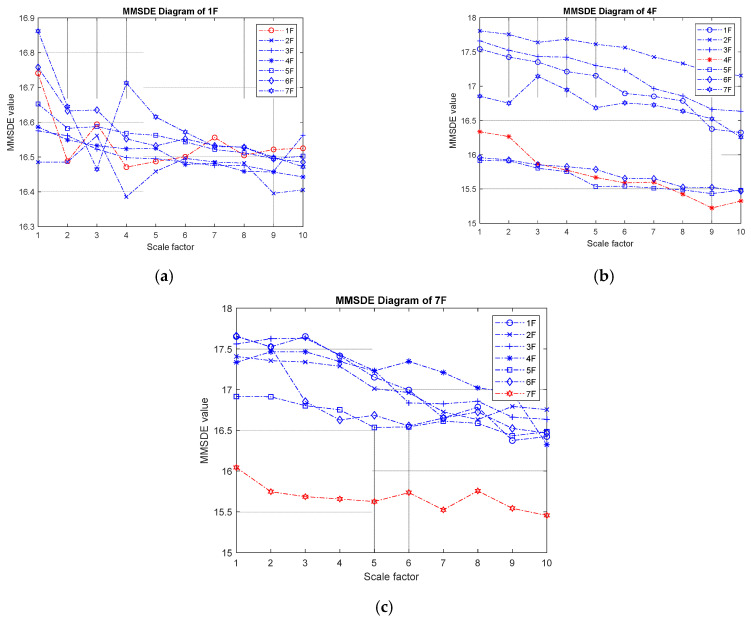
The experimental MMSDE diagrams of damage on a single story; (**a**) damage on the first floor; (**b**) damage on the fourth floor; (**c**) damage on the seventh floor.

Figure 15 shows the damaged MMSDE diagram of the single-story condition in this seven-story experimental model. In Figure 15a, the entropy curve of the damaged floor jumps up and down; therefore, the damaged floor cannot be distinguished significantly. It is presumed that this inconspicuous trend was caused by the inevitable noise signal measured by the velocity meter in the experiment. In Figure 15b, the entropy curves of the first, second, and third floors show an upward trend, as previously predicted, and the entropy curve of the fourth floor show a downward trend. The second floor MMSDE value shows a very different pattern due to the relatively high reference entropy curve established from the health condition. In the case of 7F damaged, the entropy value of the MMSDE curve reduces significantly after the bracing is removed, and the entropy values of the undamaged lower floors increased.

(II) Two-story damage

**Figure 16:**
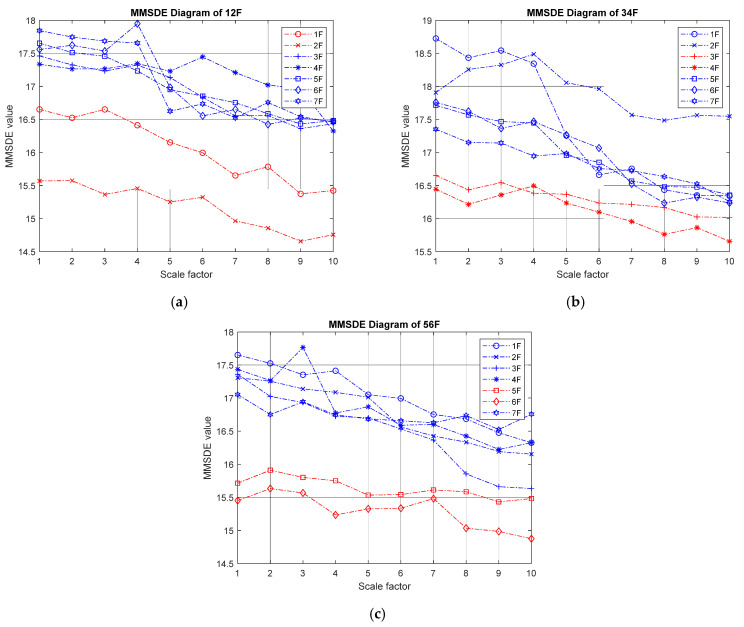
The experimental MMSEE diagram of damage on two stories; (**a**) damage on the first floor and second floor; (**b**) damage on the third floor and fourth floor; (**c**) damage on the fifth floor and sixth floor.

Figure 16 shows the damaged MMSDE diagram of damage on two stories. In the case of two-story damage, the entropy curves of healthy and damaged floors gather in the opposite direction when diagonal braces are removed, and the entropy of the damaged floors is slightly decreased under different scales. In the cases of 1 F–2 F and 2 F–3 F consecutive damage shown in Figure 16a,b, the maximum entropy value of the damaged floor reduces significantly to less than 17. In Figure 16c, the entropy curve of the damaged floor is clearly separated from the entropy curve of the healthy floor; the maximum entropy value drops below 16.

(III) Three-story damage

**Figure 17:**
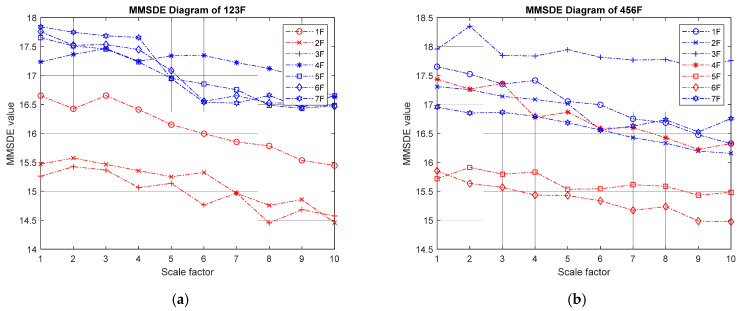
The experimental MMSDE diagram of damage on three stories; (**a**) damage on the first floor, second floor, and third floor; (**b**) damage on the fourth floor, fifth floor, and sixth floor.

**Figure 18:**
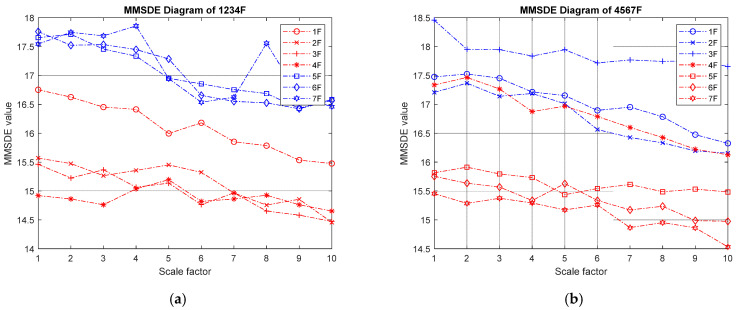
The experimental MMSEE diagram of damage on four stories; (**a**) damage on the first floor, second floor, third floor, and fourth floor; (**b**) damage on the fourth floor, fifth floor, sixth floor, and seventh floor.

In the case of a multi-story damage shown in Figure 17 and Figure 18, the entropy curves of a health floor and a damaged floor are clustered separately, and the entropy of the damaged floor decreases at different scales, which indicates that the damaged floor would be clearly distinguished. However, when the value of the MMSDE curve of the damaged floor fails to decrease as expected, the analysis of some cases becomes unstable at a high scale and causes misjudgment.

### 4.3. Damage Index

**Figure 19:**
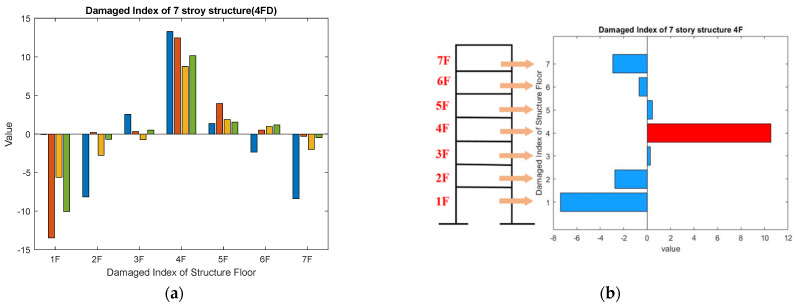
Damage index of damage on the fourth floor; (**a**) four diagnosis results; (**b**) the average diagnosis result.

When the damaged floor cannot be clearly distinguished by the MMSDE curve, the damage index (DI) is applied as a further quantitative judgment for damage detection. In the 4F damaged case, the trend of the entropy curves of 1 F, 2 F, and 3 F behave as expected. The overall entropy values increase, and the 4 F entropy value decreases, without fluctuation at high scales. The damage indices of four ambient tests and the average diagnosis result are presented in Figure 19a,b, respectively. As indicated, the destruction of the 4 F can be clearly identified from the damaged index.

**Figure 20:**
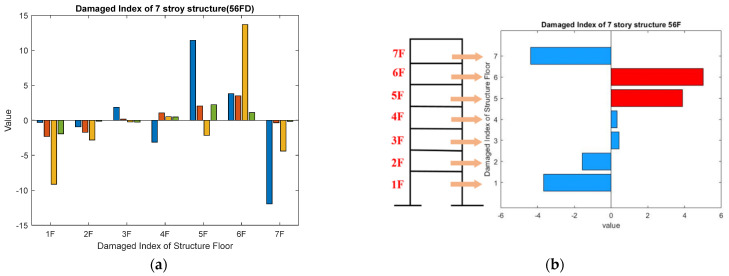
Damage index of damage on the fifth floor and sixth floor; (**a**) four diagnosis results; (**b**) the average diagnosis result.

For the case of damage on 5 F and 6 F, the curves of the damaged floor and the healthy floor are gathered separately, and the trend is significant with a low scale factor. The overall entropy of the damaged floor decreases from 16.9 to 15.5. The damage indices obtained for the damaged case of 5 F and 6 F of four ambient measurements and the average diagnosis results are presented in Figure 20. The damage on the fifth and sixth floors can be successfully identified from the damage index.

**Figure 21:**
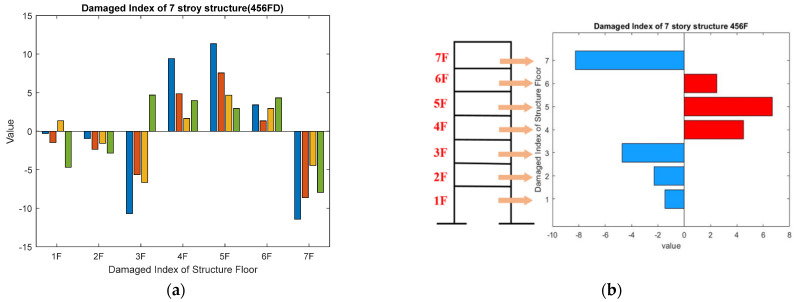
Damage index of damage from the fourth floor to the sixth floors; (**a**) four diagnosis results; (**b**) the average diagnosis result.

From the MMSDE curves for damage on floors ranging from the fourth floor to the sixth floor, the entropy curve of the fourth floor does not drop as expected. However, since the curve of the third floor rises relatively, the damage index can successfully determine the damage to the fourth floor. The entropy values of the fifth and sixth floors decrease significantly. The damage of these three floors can be successfully identified by the damage index, and the results are presented in Figure 21.

### 4.4. Confusion Matrix Verification

The damage index results were evaluated by the confusion matrix defined in Section 3.4, where the results were classified into four categories: TP, TN, FP, and FN. As shown in Table 4, the accuracy rate is 93%, and the precision rate is 88%. The strong recall rate of 85.7% indicates that most floors diagnosed as damaged are actually damaged. On the basis of the above results, the feasibility of applying MMSDE in structural health monitoring has been highly demonstrated.

According to the experimental results, the entropy curve and the damage index can accurately identify the damaged floor. In terms of the entropy curve, when a low floor is damaged, the entropy value of the adjacent floor will increase significantly with each scale. In the case of high-floor and multi-floor damage, the entropy curves of healthy and damaged floors are concentrated, respectively, and the damaged floor will slightly decrease at different scales. Moreover, by calculating the area difference of the entropy curve of adjacent floors using the damage index, the damaged floor can be determined more reliably with the quantitative results.

### 4.5. Comparison between Numerical Simulation and Experiment

From the results of numerical simulations and experiments, it is found that the entropy value of the damaged floor will increase, while that of the healthy floor will decrease. In the case of destruction to high-rise floors, the entropy value of the higher-scale floors decreases more significantly. In the case of multi-floor destruction, the curves of the healthy floor and the damaged floor are concentrated into different groups. However, in the experiment, due the inevitable noise signal and the unidentifiable interference, inconsistent detection results of single-layer damage and multi-layer damage were observed. Nevertheless, from the perspective of the three major indicators of the confusion matrix, good performance can be expected from the proposed SHM method in practical application.

## 5. Conclusions

Recently, entropy analysis has been increasingly applied to the field of civil and structural engineering. In this research, a new entropy-based method, the MMSDE analysis, is applied for structural health monitoring. In addition to comparing the MMSDE entropy curve of the undamaged and damaged cases at different scales, a damage index is proposed as a quantitative judgment for damage detection. As the SHM process is based on the ambient vibration signal, which is easy to implement, it can largely reduce the possible errors caused by human judgment and provide a reliable result.

First, the ambient vibration of a seven-story steel structure is simulated by finite element software for numerical analysis. Fifteen cases of different damage combinations are simulated. To examine the performance of the proposed method, the confusion matrix is applied. Three parameters, including accuracy, precision, and recall are systematically evaluated. As indicated, the accuracy rate is 92.8%, the precision rate is 90.3%, and the recall rate is 82.3%, respectively. The feasibility of the MMSDE method is preliminarily demonstrated.

Experimental verification was carried out on a scaled-down seven-story steel structure to validate the practical performance of MMSDE. Based on the result calculated from four ambient vibration measurements, the accuracy rate was 93%, the precision rate was 88%, and the recall rate was 82%, respectively. Although the precision rate of the experiment may drop by 2.3% due to signal interference, it still does not affect its excellent performance. In this study, numerical simulations and steel frame experiments have both clearly shown the potential to identify structural destruction. As only a slight difference was observed between the numerical simulation and the experimental verification, it strongly indicated that the MMSDE method can be effectively applied in actual buildings to identify the possible location of structural damage.

## Figures and Tables

**Table 1:** Damage degree of the numerical model.

Case Number	Damage Group	Damaged Floors
0	Undamaged	None
1		1 F
2		2 F
3		3 F
4	One-story damage	4 F
5		5 F
6		6 F
7		7 F
8		1 F and 2 F
9		2 F and 3 F
10	Two-story damage	3 F and 4 F
11		4 F and 5 F
12		5 F and 6 F
13		6 F and 7 F
14		1 F and 2 F and 3 F
15		2 F and 3 F and 4 F
16	Three-story damage	3 F and 4 F and 5 F
17		4 F and 5 F and 6 F
18		5 F and 6 F and 7 F

**Table 2:** The confusion matrix of numerical simulation.

Case	Damage	MMSDE			
Number	Floors	TP	FP	TN	FN
1	1 F	1	0	6	0
2	2 F	1	1	5	0
3	3 F	1	0	6	0
4	4 F	1	0	6	0
5	5 F	1	0	6	0
6	6 F	1	0	6	0
7	7 F	1	0	6	0
8	1 F and 2 F	1	0	5	1
9	2 F and 3 F	2	1	4	0
10	3 F and 4 F	2	0	5	0
11	4 F and 5 F	2	0	5	0
12	5 F and 6 F	2	0	5	0
13	6 F and 7 F	1	1	4	1
14	1 F and 2 F and 3 F	1	0	4	2
15	2 F and 3 F and 4 F	2	0	4	1
16	3 F and 4 F and 5 F	2	0	4	1
17	4 F and 5 F and 6 F	3	0	4	0
18	5 F and 6 F and 7 F	3	0	4	0
Total		28	3	89	6
Accuracy		92.80%			
Precision		90.30%			
Recall		82.30%			

**Table 3:** Fundamental frequency of all damage cases.

The Number of Case	Damaged Case Group	Damage Floors	Frequency (Hz)
0	Undamaged	None	3.34
1	One-story damage	1 F	2.08
2		2 F	2.13
3		3 F	2.12
4		4 F	2.29
5		5 F	2.61
6		6 F	2.88
7		7 F	3.2
8	Two-story damage	1F, 2 F	1.64
9		3 F, 4 F	1.83
10		5 F,6 F	2.32
11	Three-story damage	1 F, 2 F, 3 F	1.44
12		4 F, 5 F, 6 F	1.88
13	Multi-story damage	1 F, 2 F, 3 F, 4 F	1.33
14		4 F, 5 F, 6 F, 7 F	1.86

**Table 4:** The confusion matrix of experimental verification.

Case	Damage	MMSDE			
Number	Floors	TP	FP	TN	FN
1	1 Floor	1	0	6	0
2	2 Floor	1	0	6	0
3	3 Floor	1	0	6	0
4	4 Floor	1	0	6	0
5	5 Floor	1	0	6	0
6	6 Floor	1	0	5	1
7	7 Floor	1	1	5	0
8	1 F and 2 F	2	0	5	0
9	3 F and 4 F	2	0	5	0
10	5 F and 6 F	2	1	4	0
11	1 F and 2 F and 3 F	2	1	3	1
12	4 F and 5 F and 6 F	3	0	4	0
13	1 F and 2 F and 3 F and 4 F	2	0	3	2
14	4 F and 5 F and 6 F and 7 F	4	0	3	0
Total		24	3	67	4
Accuracy		93.8%			
Precision		88%			
Recall		85.7%			
